# Ultra-Low-Voltage CMOS-Based Current Bleeding Mixer with High LO-RF Isolation

**DOI:** 10.1155/2014/163414

**Published:** 2014-08-14

**Authors:** Gim Heng Tan, Roslina Mohd Sidek, Harikrishnan Ramiah, Wei Keat Chong, De Xing Lioe

**Affiliations:** ^1^Department of Electrical and Electronic Engineering, Universiti Putra Malaysia, 43400 Serdang, Malaysia; ^2^Department of Electrical and Electronic Engineering, Segi University, 47810 Petaling Jaya, Selangor, Malaysia; ^3^Department of Electrical Engineering, University of Malaya, 50603 Kuala Lumpur, Malaysia

## Abstract

This journal presents an ultra-low-voltage current bleeding mixer with high LO-RF port-to-port isolation, implemented on 0.13 *μ*m standard CMOS technology for ZigBee application. The architecture compliments a modified current bleeding topology, consisting of NMOS-based current bleeding transistor, PMOS-based switching stage, and integrated inductors achieving low-voltage operation and high LO-RF isolation. The mixer exhibits a conversion gain of 7.5 dB at the radio frequency (RF) of 2.4 GHz, an input third-order intercept point (IIP3) of 1 dBm, and a LO-RF isolation measured to 60 dB. The DC power consumption is 572 *µ*W at supply voltage of 0.45 V, while consuming a chip area of 0.97 × 0.88 mm^2^.

## 1. Introduction

Various low-power front-end receivers had been widely reported for application such as wireless sensor network (WSN). WSN application which requires low-power operation often adopts ZigBee standard with the operating frequency ranging from 2.4 to 2.4835 GHz [1]. Direct conversion receiver (DCR) has been in the heat of discussion in recent years due to its inherent low power consumption and the simplicity in realization [2]. One of the major setbacks associated with DCR is the LO to RF port-to-port isolation. An incurred mismatch in the device physical dimension would potentially couple the LO leakage to the RF port through the gate-drain capacitance, *C*_gd_ of the RF transconductance transistors. The leakage component will mix with RF signal, resulting in a detrimental phenomenon known as self-mixing [3] which in effect produces DC offset that degrades the performance of the overall receiver. In preference, high isolation between the LO-RF ports is crucial in alleviating self-mixing. The typical LO-RF isolations of the standard Gilbert cell mixer are in the range of 40–50 dB [4]. In this paper, the conventional current bleeding architecture which has a high conversion gain and low noise figure is modified by integrating a combination of NMOS-based current bleeding transistor, PMOS-based LO switch, and integrated inductors, thus improving the isolation between the LO and RF port.

## 2. Proposed Design

**Figure 1:**
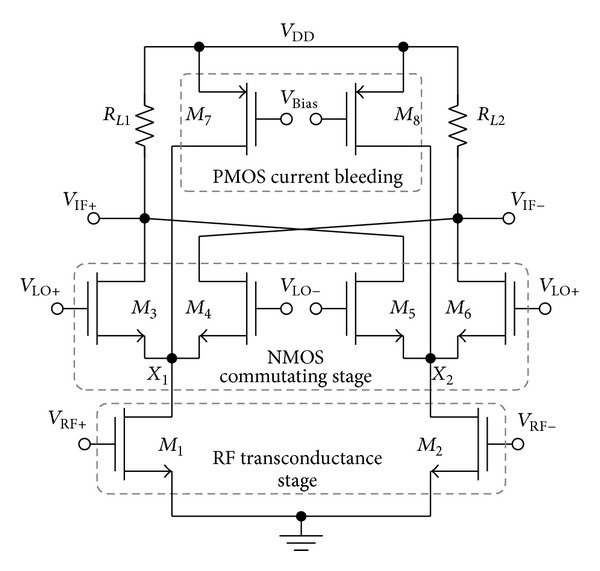
Conventional current bleeding mixer.

Previous design: Figure 1 shows the conventional CMOS current bleeding mixer that integrates a combination of PMOS-based current bleeding stage and NMOS-based local oscillator, LO switching stage [5].

In effect to the mismatch in the switching stage, the LO leakage component at nodes *X*_1_ and *X*_2_ will directly couple to the RF port through the gate-drain capacitance, *C*_gd_, of the RF transconductance stage (*M*_1_-*M*_2_). This will adversely reduce the isolation between the LO and RF ports.

**Figure 2:**
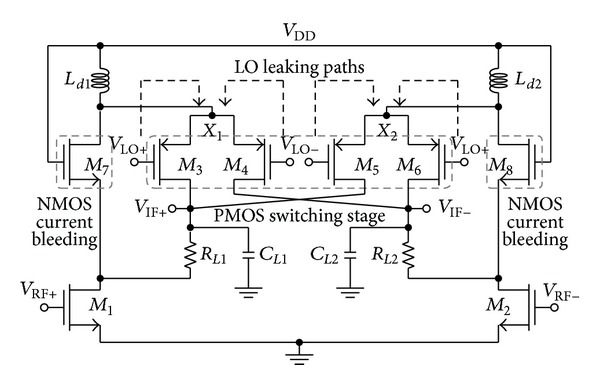
A schematic view of the proposed mixer.

Figure 2 shows that the proposed mixer consists of a RF transconductance stage (*M*_1_-*M*_2_), a PMOS-based LO switching input (*M*_3_–*M*_6_), a NMOS-based current bleeding stage (*M*_7_-*M*_8_), and the output load (*R*_*L*1_-*C*_*L*1_ and *R*_*L*2_-*C*_*L*2_). Inductors *L*_*d*1_ and *L*_*d*2_ act as a RF choke in alleviating the RF signal leakage into the voltage supply, *V*_DD_. The RF frequency is mixed with the LO frequency at node *X*_1_ and *X*_2_. The differential output current, neglecting the higher order spurs, can be derived as follows:

(1) iIF=2π·gm1,2 ·vRF[sin(ωRF−ωLO)t−sin(ωRF+  ωLO)t],

where *g*_*m*1,2_ is the transconductance for *M*_1_ and *M*_2_ and *v*_RF_ is the input RF signal, while *ω*_RF_ and *ω*_LO_ are the RF and LO frequency, respectively. At the IF output, the combination of *R*_*L*1_-*C*_*L*1_ and *R*_*L*2_-*C*_*L*2_ forms a low pass filter (LPF), which filters out the high-order spurs at the output such as the upconverted frequency component sin(*ω*_RF_ + *ω*_LO_).

An incurred mismatch in the LO switching transistor physical dimension would result in a feed-through of LO leakage at nodes *X*_1_ and *X*_2_ to the RF port as described in Figure 2. The dotted arrowhead illustrates the LO leakage path from the LO ports to nodes *X*_1_ and *X*_2_.

Other than for providing a desirable bleeding path for the DC current, transistor *M*_7_-*M*_8_ is optimized to enhance the LO-RF isolation. Transistors *M*_7_ and *M*_1_ are cascoded in series, thus observing high impedance, *R*_*X*_ referring to the drain terminal of the bleeding transistor *M*_7_, expressed as

(2) $$\begin{array}{c} R_{x}\approx g_{m7}\cdot r_{o1}\cdot r_{o7}, \end{array}$$

where *g*_*m*7_ and *r*_*o*7_ are the transconductance and output resistance for *M*_7_ while *r*_*o*1_ is the output resistance for transistor *M*_1_. Accordingly, this high impedance node minimizes the LO leakage from nodes *X*_1_ and *X*_2_ to the RF port. As a bench of comparison to the design in Figure 1, the LO leakage component at nodes *X*_1_ and *X*_2_ would directly couple to the RF port through parasitic capacitance *C*_gd_ in the absence of additional shielding between LO-RF port. The proposed mixer on the other hand utilizes the bleeding transistor (*M*_7_ and *M*_8_) as the shielding element between LO and RF port to improve the LO-RF isolation. In addition, the high frequency LO leakage at the output node of *V*_IF+_ and *V*_IF−_ as shown in Figure 2 is insignificant as it is directed towards the ground rail via the low impedance path of capacitor *C*_*L*1_ and *C*_*L*2_ in contrary to the conventional architecture where the leakage component couples to the RF port. Additionally any LO leakage at the IF output port is further attenuated by load resistor *R*_*L*(1,2)_ before reaching the RF port, in a goal of improving the LO-RF isolation.

Along with the presence of the cascoded configuration between transconductance and the bleeding stage, this mixer is able to work down to 0.45 V of supply headroom. The conventional current bleeding mixer as shown in Figure 1 requires an LO bias of

(3) $$\begin{array}{c} V_{\text{LO}}=V_{\text{gs}3}+V_{\text{ds}1(\mathrm{sat})}, \end{array}$$

where *V*_LO_ is the DC voltage to bias the switching transistors, *V*_gs3_ is the gate to source voltage of transistor *M*_3_, and *V*_ds1(sat⁡)_ is the overdrive voltage of transistor *M*_1_. By adapting PMOS-based LO switching stage (*M*_3_–*M*_6_), coupled together with inductors *L*_*d*1_ and *L*_*d*2_ as illustrated in Figure 2, the DC voltage required to bias the gate of transistor *M*_3_–*M*_6_ is reduced to only *V*_sg_ (source-gate voltage) which approximate to the threshold voltage, *V*_th_ of the PMOS transistor, whereas the DC voltage at nodes *X*_1_ and *X*_2_ approaches to *V*_DD_. The LO bias voltage *V*_LO_ is given as

(4) $$\begin{array}{c} V_{\text{LO}}=V_{\text{dd}}-V_{\text{th}3}, \end{array}$$

where *V*_th3_ is the threshold voltage of transistor *M*_3_. The DC voltage required to turn on the LO switching stage no longer depended on the overdrive voltage, *V*_ds1,2(sat⁡)_ of the transconductance stage (*M*_1_-*M*_2_) as given in (3). In this proposed mixer, the DC voltage for *V*_LO_ nears ground potential instead of the positive power rail resulting the design to operate favorably at ultra-low supply headroom. As for the conventional current bleeding mixer architecture as in Figure 1, the DC bias voltage for *V*_LO_ moves towards the positive rail providing a bottleneck in operating the mixer at low supply headroom.

## 3. Measurement Result

**Figure 3:**
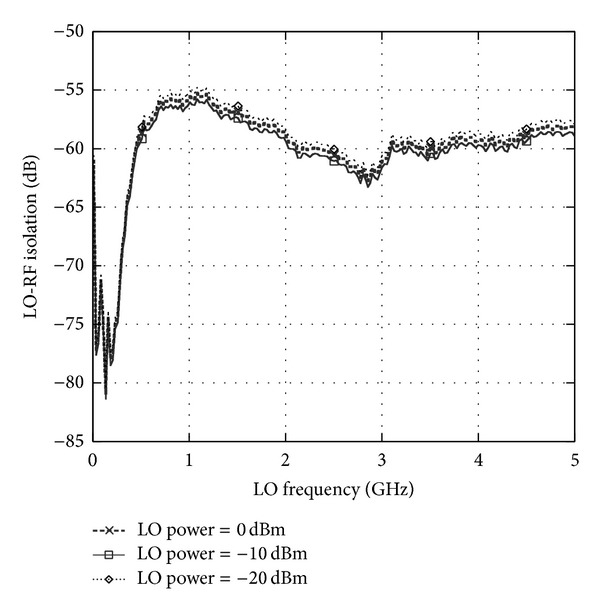
Measured LO-RF isolation.

**Figure 4:**
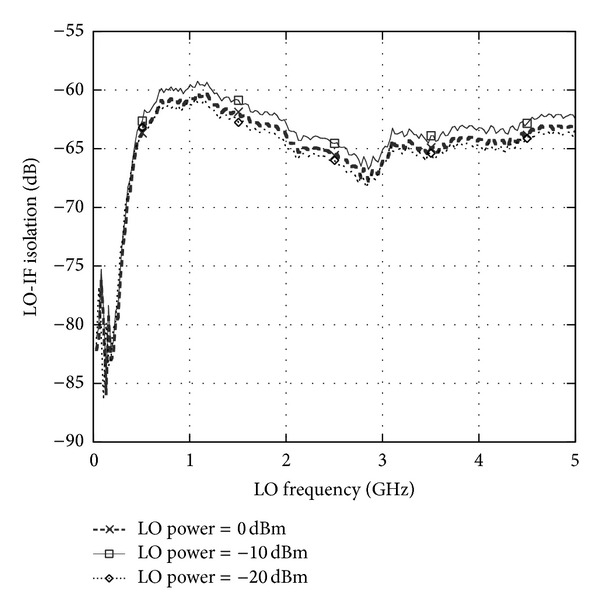
Measured LO-IF isolation.

**Figure 5:**
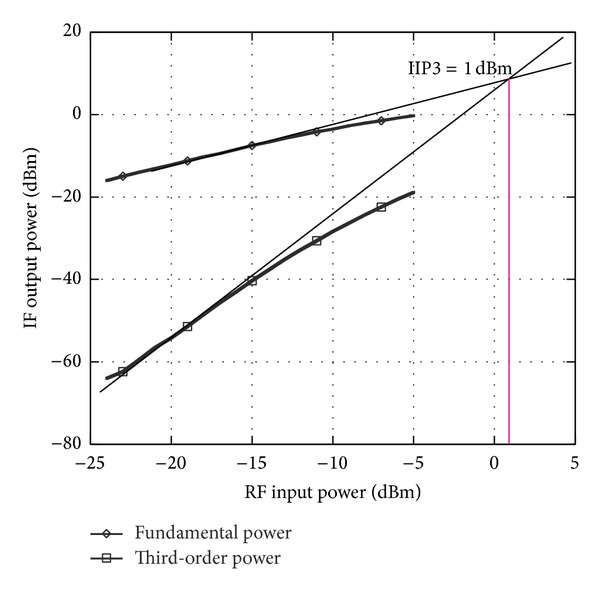
IIP3 of the proposed mixer.

**Figure 6:**
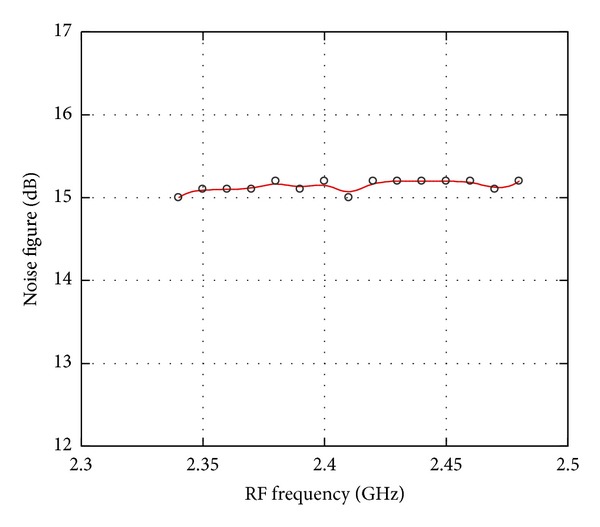
Noise figure.

**Figure 7:**
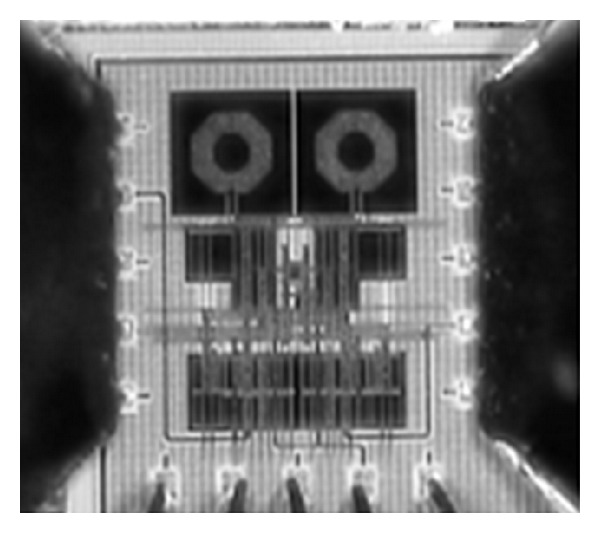
A chip micrograph of the proposed mixer.

The proposed mixer is implemented on a 0.13 *μ*m standard CMOS technology. The mixer consumes only 1.27 mA of DC current from 0.45 V of supply voltage. The LO-RF isolation in dB is given as [10]

(5) $$\begin{array}{c} P_{\text{Isolation}}\left(\text{dBm}\right)=P_{\text{flo}(\text{LO})}\left(\text{dBm}\right)-P_{\text{flo}(\text{RF})}\left(\text{dbm}\right), \end{array}$$

where *P*_Isolation_ is the isolation between LO and RF port due to the leakage component from LO port, *P*_flo(LO)_ is the injected LO power at LO port, and *P*_flo(RF)_ is the observed LO power coupled to the RF port. The LO-RF isolation has been measured at a difference discrete LO power as described in Figure 3. It can be observed that from the frequency of 2 GHz to 5 GHz, the isolation achieved is more than 55 dB and at 2.4 GHz, the LO-RF isolation is measured at 60 dB. The isolation technique adapted in this circuit has improved the LO-RF shielding significantly while operating at ultra-Low supply voltage down to 0.45 V.  Figure 4 shows the results for LO-IF isolation which measures more than 64 dB at 2.4 GHz. Two-tone test with an input frequency of 2.443 GHz and 2.442 GHz is applied to the RF port with the corresponding LO frequency of 2.439 GHz to quantify the linearity of the mixer. The conversion gain of the proposed mixer is observed to be 7.5 dB with an IIP3 of 1 dBm as shown in Figure 5. Inductors  *L*_*d*1_ and *L*_*d*2_ in the proposed mixer of Figure 2 not only function as the DC current source but concurrently resonate out the parasitic capacitance at nodes *X*_1_ and *X*_2_ to improve the IIP3. The noise figure (NF) is observed to be around 18 dB as illustrated in Figure 6.  Table 1 summarizes the design parameters for the proposed mixer and the performance comparison of the proposed architecture respective to other reported works is given in Table 2. The designed mixer has the highest LO-RF isolation and among the lowest in DC power consumption. The measured conversion gain and linearity, IIP3, of the mixer is 7.5 dB and 1 dBm, respectively. The dynamic performance of the architecture is evaluated adapting a figure of merit (FOM) expression, which is highlighted in the following equation, given as [11]:

(6) $$\begin{array}{c} \text{FOM}=10\log\left(\frac{10^{G/20}\cdot 10^{(\text{IIP}3-10)/20}}{10^{\text{NF}/10}\cdot P}\right), \end{array}$$

where *G* is the conversion gain in dB, IIP3 is the third order linearity in dBm, NF is the noise figure in dB, and *P* is the power in mW. In reasoning out the performance comparison respective to other reported recent work, the proposed architecture exhibits the highest FOM of 16.67 while relating to power dissipation well below 1 mW. In the loop of recent reported work, the proposed architecture process to be the lowest in power consumption. The photomicrograph of the chip is illustrated in Figure 7, with a corresponding chip area of 0.97 × 0.88 mm^2^.

## 4. Conclusion

The proposed mixer is successfully designed and verified in 0.13 *μ*m standard CMOS technology. The implemented CMOS-based current bleeding mixer topology, which consists of a combination of NMOS-based current bleeding transistor, PMOS-based switching stage, and integrated inductors, has significantly improved the LO-RF isolation while operating at supply voltage headroom down to 0.45 V. The design observes a considerable high LO-RF isolation of 60 dB and consuming merely 572 *μ*w of power, which is a promising performance metric for ZigBee application.

## Figures and Tables

**Table 1:** Design parameters for the mixer.

Parameters	Design values
*M*_1_, *M*_2_	106 *μ*m/0.13 *μ*m
*M*_3_, *M*_4_, *M*_5_, *M*_6_	64 *μ*m/0.13 *μ*m
*M*_7_, *M*_8_	200 *μ*m/0.13 *μ*m
*L*_*d*1_, *L*_*d*2_	6.7 nH
*C*_1_, *C*_2_	10 pF
*R*_*L*1_, *R*_*L*2_	1 kΩ

**Table 2:** Performance summary and comparison.

Parameter	This work	[6]	[7]	[8]	[9]
Supply voltage (V)	0.45	2.5	1.5	1	1
LO frequency (GHz)	2.4	2.525	2.4	2.4	2.4
CG (dB)	7.5	9.5	−3.5	15.7	5.3
IIP3 (dBm)	1	−7.5	0	−9	4.6
LO-RF isolation (dB)	60	48	16.8	33	—
NF (dB)	15	—	—	18.3	21.7
Power (mW)	0.572	17.5	2	0.5	3.5
FOM	16.67	—	—	13.06	2.81
Area (mm^2^)	0.9 × 0.8	—	—	1 × 0.8	0.4 × 0.5
CMOS technology (*μ*m)	0.13	0.18	0.35	0.13	0.18
